# Trait Plasticity, Resource Redirection and Strong Recovery Capacity Enhance *Volkameria inermis* Tolerance and Adaptation to Long-Term Foliar Salt Stress

**DOI:** 10.3390/plants15111756

**Published:** 2026-06-05

**Authors:** Weilun Ding, Kunxian Tang, Jianhui Liu, Yuanmin Sun, Shan Chen, Fei Zhang, Luchun Cai, Wenhui You

**Affiliations:** 1 School of Ecology and Environmental Sciences, East China Normal University, Shanghai 200241, China; 52273903001@stu.ecnu.edu.cn; 2 Third Institute of Oceanography, Ministry of Natural Resources, Xiamen 361005, China; 3 Key Laboratory of Marine Ecological Protection and Restoration, Ministry of Natural Resources, Xiamen 361005, China; 4 Field Observation and Research Station for Marine Ecosystems of the West Taiwan Straits Coastal Islands, Ministry of Natural Resources, Xiamen 361005, China; 5 Key Laboratory of Marine Ecological Protection and Restoration of Fujian Province, Xiamen 361005, China

**Keywords:** foliar salt stress, growth and morphology, gas exchange, chlorophyll, physiological regulation, adaptive strategies

## Abstract

Salt tolerance is a key factor limiting coastal vegetation restoration. In backshore areas, foliage is frequently exposed to salt mist and wave splash, which severely constrains plant survival and restoration outcomes. While root salt tolerance under short-term stress has been widely studied, foliar salt tolerance remains poorly understood. Here, using a self-developed experimental apparatus, we investigated the salt tolerance mechanisms of the coastal shrub *Volkameria inermis* through a long-term (159-day) foliar salt stress experiment (0–3.0% NaCl), followed by a 64-day recovery period. Field suitability was also evaluated at different coastal locations in Quanzhou Bay, Fujian Province. The results show that: (1) trait plasticity (e.g., leaf thickening), resource redirection (e.g., reduced growth rate, and new bud emergence in unstressed parts), and strong recovery capacity together enhance *V. inermis* adaptation to long-term foliar salt stress; (2) *V. inermis* exhibits adaptability to salinity ≤2.0% and survival under 3.0% despite severe injury; (3) besides osmotic adjustment, proline accumulation helps alleviate oxidative damage; and (4) field data demonstrated that leaf thickness and leaf water content were significantly associated with distance from the sea and elevation, thereby validating the salt-adaptation strategies observed under controlled conditions. This study provides a novel methodological framework and practical insights for selecting salt-tolerant species in coastal restoration.

## 1. Introduction

Coastal vegetation plays a vital role in stabilizing coastal ecosystems and enhancing landscape quality [1]. Among various abiotic stresses, salinity is a primary determinant of geographic distribution of coastal plants. Marine salts affect all plant organs (roots, stems, leaves, etc.) through multiple pathways, including seawater intrusion, wave splash, sea spray, and salt mist [2,3]. A vegetation survey in Pingtan and surrounding islands of Fujian, China, revealed that the *Volkameria inermis* community on the backshore of Tangyu Island experiences severe salt stress during winter monsoons (Supplementary Figure S1). Leaves facing the sea, exposed to salt mist and wave splash, show significant morphological adaptations such as thickening and succulence. In contrast, surrounding communities of *Ipomoea pes-caprae* and *Vitex rotundifolia* exhibited severe leaf yellowing and abscission. Similarly, in Longfengtou Bay, Pingtan, seaward branches of planted vegetation withered due to salt spray driven by winter monsoons and waves (Supplementary Figure S2). These observations indicate that foliar salt stress poses a greater constraint to plant growth in elevated coastal areas, thereby hindering the effectiveness of vegetation restoration. Consequently, this study focuses on the responses and adaptations of plant aboveground parts to salt stress.

Research has shown that osmotic stress and ion toxicity resulting from salt stress impair a plant’s ability to detoxify reactive oxygen species (ROS), leading to oxidative damage [4]. Malondialdehyde (MDA), a major product of membrane lipid peroxidation, is a widely used indicator for assessing the severity of biotic or abiotic stress [5]. Throughout evolution, plants have developed comprehensive osmotic regulation and antioxidant defense networks to enhance stress tolerance [6]. Studies have indicated that proline (Pro), a key osmotic regulator with strong hydration capacity, serves as a reliable biomarker for assessing plant stress resistance [7]. Its accumulation helps prevent protein dehydration and denaturation under osmotic stress, thereby maintaining cellular osmotic potential [8].

Photosynthesis, the fundamental process providing materials and energy for plant growth [9], is highly sensitive to environmental changes in leaves [10]. Under salt stress, alterations in stomatal behavior impede CO_2_ uptake and disrupt water transport during transpiration [11]. In severe cases, non-stomatal factors become critical [12], including disorganized chloroplast arrangement, loosening of thylakoid membranes, vacuole enlargement, and lipid body accumulation [13]. These changes damage the photosynthetic membrane system, reduce the photosynthetic pigment content [14], and ultimately inhibit photosynthesis, adversely affecting plant growth [15].

*Volkameria inermis* (L.) is a perennial evergreen climbing shrub (Lamiaceae) widely distributed along China’s southeastern coast [16]. It has the potential to treat various ailments, showing hepatoprotective, anti-inflammatory, anti-rheumatic, immunomodulatory, hypoglycemic, anti-obesity, anti-diarrheal, and anti-tranquilizing activities [17]. Research shows that its seeds fail to germinate in NaCl solutions exceeding 0.5% [18]. Under soil salinity stress, *V. inermis* accumulates Na^+^ and Cl^−^, while its chlorophyll content decreases significantly [19]. Correspondingly, proline and MDA levels increase, and antioxidant enzyme activities (e.g., superoxide dismutase activities) are enhanced [20]. Shan et al. highlighted that root epidermal cork layer thickening, increased suberin content, and lipid accumulation constitute critical barriers to salt entry into roots [21].

Most current research on plant salt tolerance has focused on crops and economically important plants [22,23,24,25]. Studies on coastal species like *V. inermis* have generally focused on short-term root salt stress (typically less than a month) [16,19,21,26], with limited studies on foliar salt stress [27]. Some studies have immersed roots in saline water for short-term observations [20,28]. However, this approach does not accurately replicate natural long-term salt stress conditions or track adaptive processes. Furthermore, we observed that leaf thickening in *V. inermis* on Tangyu Island is alleviated during rainy seasons when salt stress diminishes. Therefore, focusing solely on stress experiments fails to fully reveal the plant’s self-repair mechanisms post-damage.

To address these gaps, we selected *Volkameria inermis* as our research subject and developed the “Nested Automatic Water Circulation Experimental Device for Plant Foliar Salt Stress” [29] to mimic salt mist and wave splash. Our objectives were to: (1) observe long-term changes in plant traits (growth, morphology, photosynthesis, physiology) under controlled foliar salt stress; (2) evaluate field performance at multiple coastal locations in Quanzhou Bay, Fujian Province; and (3) assess self-recovery capacity after stress cessation. This study represents a novel methodological approach for examining salt tolerance and adaptation in specific plant organs. It provides precise guidance for species selection and configuration in coastal vegetation restoration based on regional habitat characteristics.

## 2. Materials and Methods

### 2.1. Biological Material

One-year-old seedlings of *Volkameria inermis* L., cultivated under controlled conditions in a ventilated greenhouse at the Zhangzhou Gulei Experimental Base, were selected as experimental material. Forty uniform and healthy seedlings were transplanted into planting bags (10 cm diameter × 10 cm height) containing a 1:1 mixture of organic fertilized soil and natural soil. Plants were acclimated in the greenhouse for two weeks before the experiment.

### 2.2. Experimental Design

Based on field surveys and the varying degrees of foliar salt stress encountered in ecological restoration, four salinity levels (0.0%, 1.0%, 2.0%, and 3.0% NaCl) were established, with the 0.0% group serving as the control. The experiment was arranged as a completely randomized design. All biological material was randomly divided into several groups, with 10 seedlings in each group. Treatments were then implemented by group. This design guarantees that each experimental unit has an equal opportunity to receive any treatment, free from the influence of the experimenter’s subjective tendencies. Saltwater solutions were prepared by mixing seawater and groundwater, with salinity verified and adjusted using a salinometer (WTW Multi 3320, Xylem Inc., Washington, DC, USA). These concentrations represent the salinity of the southeastern coastal waters of China and estuarine waters, respectively.

**Figure 1:**
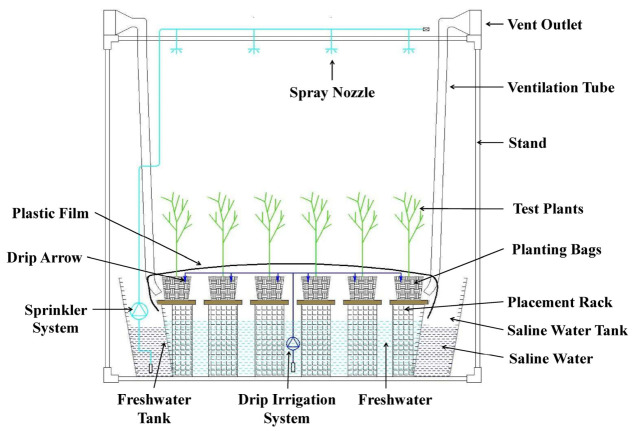
Design of a nested automatic water circulation experimental device for plant foliar salt stress.

A self-designed “Nested Automatic Water Circulation Experimental Device for Plant Foliar Salt Stress” was used to simulate different foliar salt stress habitats, which is authorized as a national invention patent [29]. Four sets of the device, identical in size and material, were established according to the treatments. Each device comprised a stand, saline and freshwater tanks, a placement rack, drip and sprinkler irrigation systems, a ventilation system, and plastic film. The configuration is illustrated in Figure 1.

The saline water tank was positioned within the stand, with the freshwater tank nested inside. The perforated placement rack, holding the test plants, was positioned above the freshwater tank. A plastic film, separating leaves from the root system, was placed above the freshwater tank and sealed tightly around the plant stem with waterproof tape.

Saline water from the respective tank was sprayed evenly onto the leaves via a sprinkler system (nozzles suspended from the stand) every 12 h (twice daily) for 1 min each. Freshwater from the nested tank was supplied to the roots via a drip irrigation system twice per week (5 min each) to maintain normal growth. Excess water draining from the planting bags flowed back into the freshwater tank through the perforated rack.

A ventilation tube, connected below the plastic film and fixed at the top of the stand, enabled air exchange between the root zone and the external environment. The outer side of the stand was enclosed with plastic film, extending into the saline tank to form a semi-enclosed space, preventing mist splash and allowing saline water recovery. All tanks were marked with water-level scales and refilled promptly when levels dropped to the minimum.

The experiment was conducted from December 2021 to August 2022. Following 159 days of foliar salt stress, the treatments were terminated, and irrigation was resumed for an additional 64-day recovery period. The four experimental devices described above were all placed in a ventilated greenhouse at the Zhangzhou Gulei Experimental Base. The greenhouse was 27 m long, 6 m wide, and had an arch height of 3.9 m.

According to data from our meteorological station (Dynamet 6) at the Gulei base in Zhangzhou, we used solar radiation greater than 0.1 W/m^2^ as the criterion for calculating sunshine duration. The calculated average sunshine duration was approximately 12.63 h in spring, 13.21 h in summer, 11.83 h in autumn, and 11.04 h in winter, with an annual average of 12.3 h. On sunny mornings between 8:00 and 11:00, the light intensity inside the greenhouse fluctuated between 800 and 1200 μmol·m^−2^·s^−1^. During the experiment, the external temperature ranged from 10 to 35 °C, while the temperature inside the greenhouse was slightly higher than the ambient temperature, and the humidity was not significantly different from that outside.

### 2.3. Measurement Indicators and Methods

#### 2.3.1. Measurement of Plant Growth and Morphological Traits

During the experiment, a quantitative scoring method was used to assess plant growth status every 7 days (Table 1) [1]. Morphological changes in leaves were recorded simultaneously. A threshold score of 2 was defined; plants scoring below this value were considered irreversibly damaged. The number of leaves per plant (LN) was recorded before, during, and after the foliar salt stress period. Leaf thickness (LT) at the same leaf position was measured using a micrometer (accuracy 0.001 mm). Soil moisture content (SMC) was monitored with the SU-LFH High-Intelligence Soil Environment Testing and Analysis Evaluation System. At the end of the stress period, root length (RL) was measured using a tape measure (accuracy 0.1 cm).

#### 2.3.2. Gas Exchange and Chlorophyll Measurements

Photosynthetic parameters were measured at regular intervals during the salt-stress period and after irrigation resumed. Each measurement was conducted during the same time period on sunny mornings. Leaves at the same position on the plants were selected from each group (at least three leaves per treatment).

Under natural conditions, the optimal temperature for photosynthesis is approximately 25 °C. When photosynthetically active radiation exceeds 1000 μmol·m^−2^·s^−1^, the net photosynthetic rate of *V. inermis* leaves increases, although the rate of increase slows under further light enhancement [30]. Based on this, a Li-6800 Portable Photosynthesis and Chlorophyll Fluorescence Measurement System (Li-Cor Inc., Lincoln, NE, USA) was employed, with leaf-chamber light intensity set to 1000 μmol·m^−2^·s^−1^, temperature to 25 °C, and relative humidity to 50%. After readings stabilized, net photosynthetic rate (Pn), transpiration rate (E), and stomatal conductance (Gsw) were recorded for each leaf. Each parameter was measured three times per leaf.

Chlorophyll (Chl) content was determined spectrophotometrically [31]. Fresh leaves were washed with distilled water, blotted dry, and the midrib was removed. The lamina was cut into small pieces, and fresh weight (m, g) was recorded. Samples were ground in a mortar with fine quartz sand and calcium carbonate powder under dark conditions. The homogenate was transferred to a 50 mL centrifuge tube, and the volume was adjusted to 40 mL (V) with an extraction solution (absolute ethanol: acetone = 1: 2, *v*/*v*). After shaking, the mixture was kept in the dark for approximately 12 h. A 3.5 mL aliquot of the supernatant was placed in a 5 mL glass cuvette, with the extraction solution as blank. Absorbance was measured at 663 nm and 645 nm (A_663_ and A_645_). Chlorophyll content was calculated as:

(1) $$Chl\;\left(mg/g\right)=\frac{\left(20.21\times A_{645}+8.02\times A_{663}\right)\times V}{m\times 1000}$$

#### 2.3.3. Measurement of Key Physiological Parameters

Proline content in leaves was determined using the acidic ninhydrin method [32]. The acidic ninhydrin reagent was prepared as a 2.5% (*w*/*v*) solution in a mixture of glacial acetic acid and 6 mol L^−1^ phosphoric acid (3:2, *v*/*v*). Leaf samples were washed with deionized water, blotted dry, and weighed (m, g). Each sample was ground in a mortar with 3% sulfosalicylic acid (extraction solution) and a small amount of quartz sand. The slurry was transferred to a centrifuge tube, and the mortar was rinsed with additional 3% sulfosalicylic acid. The combined volume was adjusted to 10 mL (V) with the same solution, then heated in a boiling water bath for 10 min. After cooling, the mixture was centrifuged at 3000 rpm for 10 min. A portion of the supernatant was transferred to a test tube. For color development, 2 mL of supernatant was mixed with 2 mL of glacial acetic acid and 2 mL of acidic ninhydrin reagent, heated in a boiling water bath for 40 min, cooled to room temperature, and extracted with toluene. The absorbance of the toluene phase was measured at 520 nm. A standard curve was prepared using proline solutions (0–10 μg mL^−1^) treated identically. Proline content was calculated as:

(2) $$Pro\;\left(ug/gFW\right)=\rho \times V÷m$$

where *ρ* is the proline concentration (μg·mL^−1^) obtained by substituting A520 into the regression equation of the standard curve, *V* is the total extract volume (mL), and *m* is the fresh weight of the leaf sample (g).

Malondialdehyde (MDA) content was measured using the thiobarbituric acid (TBA) method [33]. Leaf samples were weighed (m, g), ground with 2 mL of 10% trichloroacetic acid (TCA) and quartz sand, and the volume was adjusted to 10 mL (V) with TCA. After centrifugation (4000 r/min, 10 min), 2 mL of supernatant was mixed with 2 mL of 0.6% TBA (prepared in 10% TCA). The mixture was heated in a boiling water bath for 15 min, cooled, and centrifuged. Absorbance was measured at 532 nm, 600 nm, and 450 nm (A_532_, A_600_ and A_450_). MDA concentration was calculated as:

(3) $$C_{measured}\;\left(umol/L\right)=\left(A_{532}-A_{600}\right)\times 6.45-0.56\times A_{450}$$

(4) $$MDA\;\left(umol/g\right)=C_{measured}\times V÷m$$

### 2.4. Suitability Assessment of Volkameria inermis for Coastal Vegetation Restoration

Based on meteorological data and surveys for Jinjiang City, seawater salinity in Quanzhou Bay ranges from 20‰ to 23‰. The region has a distinct monsoon climate, with prevailing north winds in winter and south winds in summer. Environmental stressors such as typhoons, storm surges, salt mist, low winter temperatures, and strong winds significantly influence coastal vegetation. In May 2022, uniform *V. inermis* plants were selected from the marine ecological restoration project area in the Jinjiang section of Quanzhou Bay and planted at five coastal locations to assess the species’ adaptability to different coastal habitats: (I) Exposed mudflats in high-tide-zone mangrove clearings, (II) Toe of seawall slope affected by spring tides, (III) Toe of seawall slope adjacent to mature mangrove margins, (IV) Toe of seawall slope without mature mangrove forest ahead, (V) Top of seawall slope without mature mangrove forest ahead.

Geographic location and elevation of planting sites were measured using a total-station RTK (CHC V100). Data were processed with Trimble software (v12.0.0) and plotted in the CGCS2000 coordinate system. Offshore distances were calculated using Google Maps (v7.3.4.8428) satellite imagery.

After two years, a follow-up survey was conducted. Plant growth status and leaf morphology were evaluated as described in Section 2.3.1. Fresh leaves were collected, weighed (Mf), dried at 65 °C to constant weight, and re-weighed (Md) after cooling in a desiccator. Leaf water content (LWC) was calculated as:

(5) $$LWC\;(\%)=\frac{\left(Mf\;-\;Md\right)\times 100\%}{Md}$$

### 2.5. Data Analysis

All data were subjected to analysis of variance (ANOVA) using SPSS 25.0. Means were compared using Fisher’s least significant difference (LSD) test at *p* < 0.05 and *p* < 0.01 to identify significant differences among treatments. Because the growth status value (0–5) is ordinal, differences among treatment groups were analyzed using the Kruskal–Wallis test, followed by Dunn’s test for multiple comparisons. Statistical significance was set at *p* < 0.05 and *p* < 0.01. Pearson correlation analysis was performed to evaluate relationships between morphological, physiological, and photosynthetic parameters. All figures were generated using Microsoft Excel.

The “letter method” was used to denote significant differences among means. Different capital letters above the bars indicate extremely significant differences at *p* < 0.01, while different lowercase letters indicate significant differences at *p* < 0.05.

## 3. Results

### 3.1. Foliar Salt Stress Experiment in Greenhouse

#### 3.1.1. Soil Moisture Content

**Figure 2:**
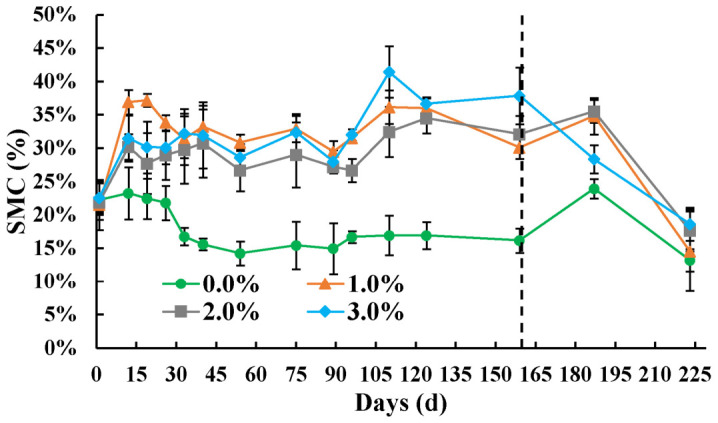
Changes in soil moisture content in the planting bags of *Volkameria inermis* under the same scheduled and quantifiable drip irrigation treatment for the roots during the experiment. Note: The vertical dashed line in the figure marks the boundary between the foliar salt stress period and the irrigation period.

Before the experiment began, SMC in the planting bags remained at around 22% across all treatments (Figure 2). After foliar salt stress was initiated, SMC in the control group gradually decreased to about 15% and then fluctuated near this level. Such stable fluctuation suggested the reliability of the experimental device. In contrast, SMC in the salt-stressed groups increased progressively compared with the control, showing significant differences from day 12 (*p* < 0.05) and highly significant differences from day 26 (*p* < 0.01). Subsequently, SMC in each stressed group fluctuated between 27% and 40%, with no significant differences among the groups.

**Figure 3:**
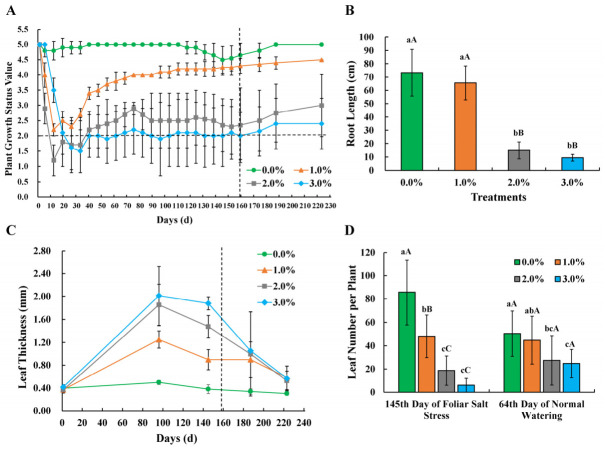
Changes in growth and morphological traits of *Volkameria inermis* during the experiment. (**A**) Plant growth status value; (**B**) Root length (RL) at the 159th day of foliar salt stress; (**C**) Leaf thickness (LT); (**D**) Average number of leaves per plant (LN). Note: The vertical dashed line represents the boundary between the foliar salt stress period and the irrigation period. The horizontal dashed line indicates the critical value for plant growth status. In Figures (**B**,**D**), different capital letters above the bars indicate extremely significant differences at *p* < 0.01, while different lowercase letters indicate significant differences at *p* < 0.05.

**Figure 4:**
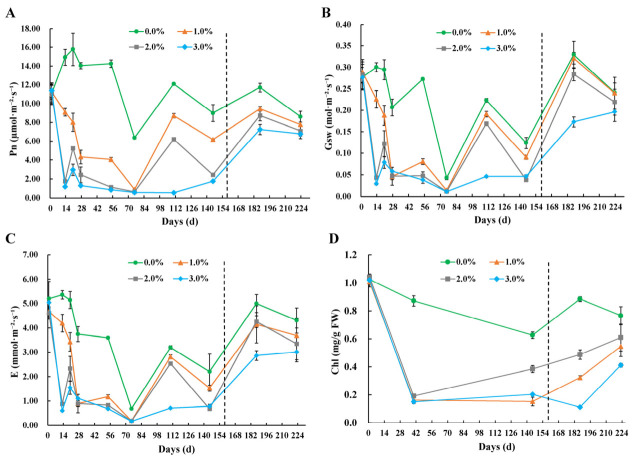
Changes in photosynthetic traits of *Volkameria inermis* leaves during the experiment. (**A**) Net photosynthetic rate (Pn); (**B**) Stomatal conductance (Gsw); (**C**) Transpiration rate (E); (**D**) Chlorophyll content (Chl). Note: The vertical dashed line represents the boundary between the foliar salt stress period and the irrigation period.

Throughout the experiment, planting bags and stem bases were shielded from salt spray by plastic film and received regular freshwater drip irrigation. Thus, evaporation had little influence on SMC variation. Instead, the decline in plant growth and development (Figure 3, Table 1), especially in photosynthesis and transpiration (Figure 4A–C), under foliar salt stress markedly affected SMC. After stress cessation and the resumption of irrigation, growth and photosynthetic activity recovered gradually in the stressed groups, and SMC correspondingly decreased to levels not significantly different from the control.

#### 3.1.2. Growth and Morphology

Growth and morphological changes are direct indicators of plant stress responses. The scoring method effectively quantifies growth status. Before the experiment, all *V. inermis* plants were healthy, with uniformly green leaves of similar thickness (Figure 3A,C). During the first 14 days of foliar salt stress, leaves in the stressed groups showed color fading, marginal wilting, desiccation, and eventual yellowing and shedding of older leaves, with severity increasing with salt concentration. Plant growth status declined rapidly in all stressed groups compared to the control, showing significant differences from day 5 (*p* < 0.05) and highly significant differences from day 12 (*p* < 0.01). Although apical bud necrosis inhibited vertical growth, no plant died at this stage. The 1.0% and 2.0% groups reached their poorest growth status earlier, while the 3.0% group declined more slowly, possibly due to a temporary stress tolerance mechanism induced by high salinity.

In the following 14 days, growth status in the 3.0% group continued to decline, reaching its lowest point on day 28. In the 1.0% and 2.0% groups, extensive senescence and abscission of older leaves occurred alongside the emergence of new buds along stems. New leaves were lighter in color than the control but showed significantly greater thickness and succulence as salt concentration increased (*p* < 0.01, Figure 3C). From the fifth week onward, growth status recovered gradually in all groups, with the 1.0% group recovering fastest. By the end of the stress period (day 159), growth status in the 1.0% group was not significantly different from the control, suggesting adaptability to this salt concentration. The growth status value in the 2.0% group remained above 2.5, suggesting tolerance to this concentration. In the 3.0% group, growth status fluctuated around the critical value of 2, suggesting that plants survived despite severe injury caused by this high salinity.

At the end of the stress period, the average number of leaves per plant (LN) decreased significantly with increasing salt concentration (*p* < 0.01, Figure 3D). Abscission of older leaves removes salt that had entered through leaf surfaces, while stress-induced growth inhibition reduced new leaf production, which in turn might limit further salt entry via leaves. Developing and maintaining thicker leaves represents an adaptive response to the foliar salt stress. RL was significantly shorter in the 2.0% and 3.0% groups than in the control (*p* < 0.01, Figure 3B), but not in the 1.0% group. This suggests that prolonged high-concentration foliar salt stress inhibits root development, whereas sustained low-concentration exposure has minimal impact.

After irrigation resumed, stressed plants gradually regreened. Although morphological differences from the control remained, LT and LN showed recovery trends, and previously necrotic branches produced new buds, indicating strong self-recovery capacity. Correspondingly, the plant growth status value continued increasing. After 64 days of irrigation, two plants in the 2.0% and 3.0% groups died (90% survival), while all plants in the 1.0% and control groups survived (100% survival).

#### 3.1.3. Photosynthesis

During the first four weeks of foliar salt stress, Gsw of *V. inermis* leaves decreased sharply with increasing salt concentration. Stomatal closure was accompanied by reduced E and suppressed Pn (Figure 4A–C). On day 20, the differences in Gsw, E, and Pn among treatments were highly significant (*p* < 0.01). From week 5 to week 11, the differences in these parameters among the stress groups gradually narrowed, but these parameters remained significantly lower than those of the control (*p* < 0.01). Thereafter, toward the end of the stress period (day 145), Gsw, E, and Pn in the 1.0% group increased gradually to levels not significantly different from the control. In the 2.0% and 3.0% groups, the gap between these parameters and the control also decreased, but these parameters remained significantly lower (*p* < 0.01).

**Figure 5:**
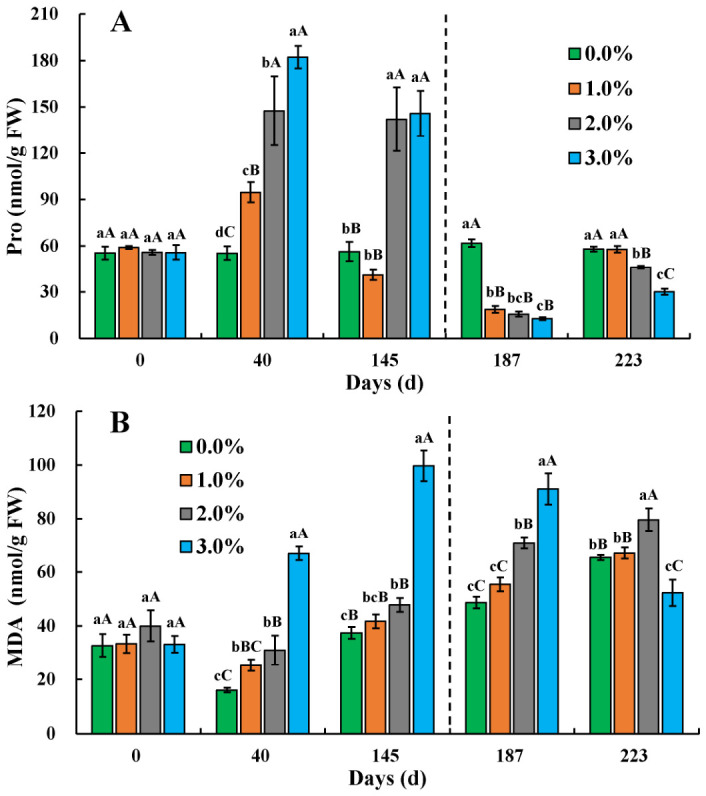
Changes in proline and malondialdehyde content of *Volkameria inermis* leaves during the experiment. (**A**) Proline; (**B**) Malondialdehyde. Note: The vertical dashed line represents the boundary between the foliar salt stress period and the irrigation period. Different capital letters above the bars indicate extremely significant differences at *p* < 0.01, while different lowercase letters indicate significant differences at *p* < 0.05.

Foliar salt stress also significantly reduced leaf chlorophyll content (Figure 4D). Before the experiment, Chl content was similar across all groups. During the first four weeks of stress, Chl in the stressed groups decreased by as much as 80% compared with the control (*p* < 0.01), contributing to the reduction in net photosynthetic rate through non-stomatal factors. On day 145, Chl content in the 2.0% and 3.0% groups was 152% and 33% higher, respectively, than that in the 1.0% group (both *p* < 0.01), suggesting that moderate to high salinity may partially stimulate chlorophyll synthesis. However, oxidative damage reflected by MDA accumulation (Figure 5B) ultimately kept Chl content in all stress groups significantly below the control level (*p* < 0.01). Overall, these changes suggest that *V. inermis* exhibits adaptability to low-concentration foliar salt stress and tolerance to high-concentration stress.

After stress cessation and the resumption of irrigation, Gsw, E, Pn, and Chl still showed significant differences among groups (*p* < 0.05), but overall recovered to more than 70% of the control levels. This suggests that once salt deposition on leaves ceases, the inhibition of photosynthesis caused by both stomatal and non-stomatal factors is highly reversible.

#### 3.1.4. Proline and Malondialdehyde

Throughout the experiment, Pro and MDA contents in leaves of all stressed groups showed an initial increase followed by a decline (Figure 5). During the early stage of foliar salt stress (≤40 days), Pro content levels in the 1.0%, 2.0%, and 3.0% groups were 71%, 166%, and 229% higher than that of the control, respectively, with highly significant differences among groups (*p* < 0.01). Similarly, MDA content increased by 57%, 92%, and 273% compared to the control, indicating stronger osmotic stress and oxidative damage under short-term high-salinity exposure. At this stage, significant osmotic adjustment occurred in the leaves.

After 145 days of stress, Pro and MDA contents still rose with increasing salinity. However, in the 1.0% group, Pro and MDA levels were close to those of the control, with no significant difference. In contrast, the difference in MDA content between the 3.0% group and the control widened progressively. Together with growth and photosynthetic data, these results suggest that under prolonged low-concentration stress, *V. inermis* shifted from damage symptoms to adaptive regulation, whereas high-concentration stress continued to cause severe membrane injury.

After stress cessation and the start of irrigation (day 187 of the experiment), Proline content in stressed plants dropped significantly below the control level (*p* < 0.01), while MDA recovered to control values. Considering the relatively high soil moisture content (Figure 2) and still-depressed Gsw, E and Pn (Figure 4A–C) at that time, this pattern suggests that osmotic stress was rapidly alleviated after salt spray ended. However, growth and photosynthesis remained reduced, placing plants in a state of relative water surplus under the same irrigation regime, thus lowering the need for Pro for osmotic adjustment. As irrigation continued and physiological activity gradually recovered, Pro content in each group approached control levels, with the extent of recovery mirroring that of growth and photosynthesis. In later stages of recovery, MDA in the 3.0% group fell slightly below the control, as damaged older leaves had been replaced by new ones at the time of measurement.

#### 3.1.5. Comparison of Leaves from Plants’ Different Positions Under Foliar Salt Stress

During foliar salt stress, new branches and buds emerged at the base of the main stems in the 2.0% and 3.0% groups, where they were shielded from direct salt exposure by the protective film. In some plants, leaves above the film wilted completely, while new buds beneath the film continued to develop (Supplementary Figure S4). This response was more pronounced under higher salt concentrations. This suggests that *V. inermis* can maintain growth in unstressed tissues when other parts are subjected to salt stress, reflecting a resource-redirection strategy that enhances stress tolerance.

**Figure 6:**
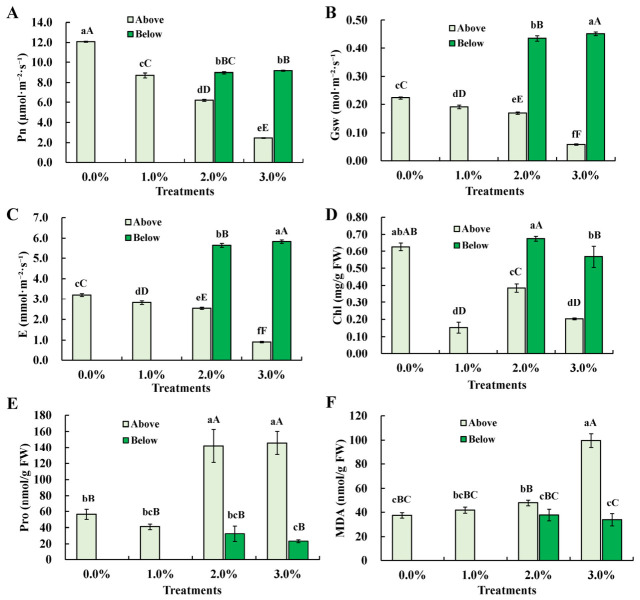
Comparison of physiological and photosynthetic traits in leaves from different positions of *Volkameria inermis* at day 145 of foliar salt stress. (**A**) Net photosynthetic rate (Pn); (**B**) Stomatal conductance (Gsw); (**C**) Transpiration rate (E); (**D**) Chlorophyll content (Chl); (**E**) Proline; (**F**) Malondialdehyde. Note: Different capital letters above the bars indicate extremely significant differences at *p* < 0.01, while different lowercase letters indicate significant differences at *p* < 0.05.

Leaves developed under the film were similar in color to the control but generally smaller and thinner. On day 145 (end of the stress period), leaves beneath the film in the 2.0% and 3.0% groups had significantly higher Pn and chlorophyll content (approximately twice as high, *p* < 0.01), and significantly higher Gsw and E (approximately twice and more than three times as high, respectively, *p* < 0.01), compared to leaves exposed to salt stress on the same plant. Meanwhile, MDA and Pro contents of leaves beneath the film were significantly lower, approaching control levels (*p* < 0.01) (Figure 6). These differences suggest minimal membrane damage and preserved integrity of photosynthetic pigments.

The lower light intensity beneath the film (due to covering and mist attachment) reduced Pn relative to the control, though it remained higher than in the 1.0% group. However, the slightly elevated temperature and humidity under the film increased stomatal aperture, resulting in Gsw and E that were significantly higher (more than twice the control, *p* < 0.01) [34], which promoted root water uptake and lowered internal osmotic potential [35]. Consequently, less proline accumulation was required for osmotic adjustment, explaining the slightly lower Pro content in leaves beneath the film compared to the control.

#### 3.1.6. Comparison of New and Old Leaves After Irrigation

**Figure 7:**
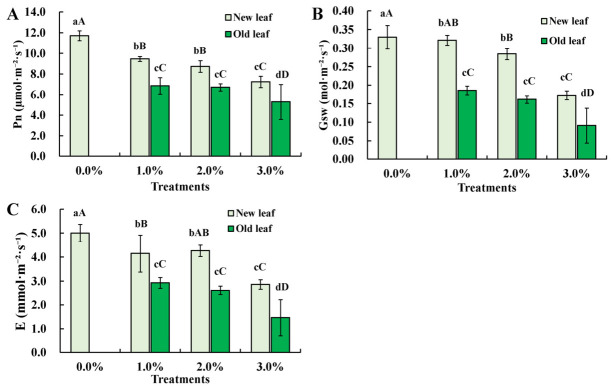
Comparison of photosynthetic traits between new and old leaves of *Volkameria inermis* after 28 days of irrigation. (**A**) Net photosynthetic rate (Pn); (**B**) Stomatal conductance (Gsw); (**C**) Transpiration rate (E). Note: Different capital letters above the bars indicate extremely significant differences at *p* < 0.01, while different lowercase letters indicate significant differences at *p* < 0.05.

After stress cessation and the resumption of irrigation, two main responses were observed: previously thickened leaves gradually became thinner, and numerous new buds emerged, with newly developed leaves showing morphology (color and thickness) closer to the control. Photosynthetic parameters of newly matured leaves and recovered older leaves were measured after 28 days of irrigation (Figure 7). Overall, the differences in photosynthetic parameters between the stressed groups and the control were substantially reduced compared to those during the stress period, suggesting a trend of recovery. In all stressed groups, Pn, Gsw, and E of new leaves were significantly higher than those of recovered old leaves (*p* < 0.01), with the differences generally within two scale units on the vertical axis. This indicates that following prolonged foliar salt stress, new leaves regain photosynthetic function more rapidly than older leaves.

Nevertheless, both new and old leaves still had significantly lower Pn, Gsw, and E values compared with the control (*p* < 0.01), and these values declined with increasing prior salt concentration. In the 3.0% group, each photosynthetic index reached only about half of the control level. This suggests that the degree of leaf damage and the subsequent recovery rate depend on the intensity of the preceding salt stress.

#### 3.1.7. Correlation Analysis Between Indices

Pearson correlation analysis (Table 2) revealed that foliar salt application rapidly and significantly affected the growth and development of *V. inermis*. Growth status showed a significant negative correlation with salt concentration. Correspondingly, several morphological and physiological traits exhibited strong correlations with salinity: LT, Pro, and MDA content were positively correlated, whereas LN, RL, Pn, Gsw, E, and Chl were negatively correlated with salt concentration.

Under prolonged stress, MDA content increased significantly with stress duration, while Gsw and E decreased over time. Both Gsw and E were highly positively correlated with Pn. Notably, growth status was positively correlated with stress duration in the later stages, indicating gradual ecological acclimation to sustained salt exposure. Under consistent root irrigation, growth status was negatively correlated with SMC, suggesting that SMC variation was driven primarily by plant growth and transpiration.

Leaf morphological traits influenced photosynthetic performance: LN and LT were significantly correlated with Pn, Gsw, E, and Chl. Both Pro and MDA were highly negatively correlated with these photosynthetic parameters, implying that sustained osmotic stress led to ionic imbalance, oxidative damage, and metabolic disruption, ultimately inhibiting photosynthesis. The strong positive correlation between Pro and MDA suggests that proline, acting as a singlet oxygen (^1^O_2_) scavenger alongside other antioxidant systems, contributed to ROS detoxification [36].

### 3.2. Growth and Leaf Traits Changes of V. inermis at Different Coastal Locations

Data from the Jinjiang Meteorological Station indicate that Quanzhou Bay has a distinct monsoon climate, with prevailing north winds in winter and south winds in summer. *V. inermis* grown at different coastal sites along the bay experienced varying levels of salt stress, with greater stress occurring at sites closer to the sea and at lower elevations. During high tide, roots were more exposed to seawater intrusion, and leaves received more wave splash.

Field survey data after two years of planting (Table 3) show that *V. inermis* survived at all locations, demonstrating broad adaptability to coastal habitats. Plants at the toe of the seawall slope were taller than those at the top, suggesting better adaptation to the slope base despite stronger salt stress, likely due to higher soil moisture content. In the high-tide zone (Location 1), leaves were significantly thicker, consistent with the salt-adaptive leaf thickening observed in controlled experiments.

**Table 3:** Summary of Survey Data for *Volkameria inermis* at Different Coastal Locations.

Location	Distance (m)	Elevation (m)	Plant Height (cm)	Crown Diameter (cm)	LT (mm)	LWC (%)
Exposed mudflats in the mangrove seedling clearings in the high tide zone	148.2	3.36 ± 0.00 eE	98.6 ± 1.5 abA	314 ± 20.6 aA	1.117 ± 0.159 aA	85.17 ± 0.58 aA
Toe of the seawall slope affected by spring tides	8.5	4.40 ± 0.03 dD	87.8 ± 19.3 abA	128 ± 38.8 cC	0.455 ± 0.127 cBC	81.96 ± 0.52 dC
Toe of the seawall slope adjacent to mature mangrove forest margins	7.8	4.75 ± 0.04 cC	106.7 ± 9.4 aA	202 ± 20.9 bB	0.611 ± 0.151 bB	83.72 ± 0.84 bB
Toe of the seawall slope without mature mangrove forest ahead	16.0	5.16 ± 0.06 bB	108.7 ± 31.2 aA	231 ± 38.9 bB	0.466 ± 0.061 cBC	82.76 ± 0.55 cC
Top of the seawall slope without mature mangrove forest ahead	6.0	6.08 ± 0.01 aA	69.8 ± 15.5 bA	80 ± 12.4 cC	0.397 ± 0.063 cC	79.22 ± 1.16 eD

Note: Different capital letters after all data indicate extremely significant differences at *p* < 0.01, while different lowercase letters indicate significant differences at *p* < 0.05.

LT and LWC differed significantly among sites (*p* < 0.01), reflecting the impact of habitat heterogeneity on plant morphology. Pearson correlation analysis (Table 4) revealed that LT and LWC were highly negatively correlated with elevation and positively correlated with each other. This suggests that lower elevations and closer proximity to the coast intensify the effects of salt mist and wave splash on leaves. The coordinated increase in LT and LWC appears to be a self-regulatory strategy for managing internal ion concentrations under salt stress.

## 4. Discussion

### 4.1. Sensory and Rapid Response at the Beginning of Stress

This study shows that within the initial two weeks of foliar salt stress, the growth status in all stressed groups decreased rapidly with increasing salt concentration. Morphologically, older leaves exhibited salt injury symptoms earlier than new leaves, beginning at the leaf tips and margins and progressing to complete chlorosis, desiccation, and abscission. Concurrently, apical bud development was inhibited and gradually turned brown. These observations are similar to the growth responses of many plants, including *V. inermis*, under short-term root salt stress [19,20,28,37]. Thus, whatever the kind of salt stress is, the most direct plant response in the short term is growth inhibition.

Studies on root salt stress in *V. inermis* have shown that osmotic stress induced by salt ions causes cell dehydration and physiological drought [37]. The accumulation of proline in leaves alleviates the osmotic stress caused by salt ions invading the aboveground parts of the plant [10]. When saltwater was sprayed directly onto the surface of the leaves, proline content in all stressed groups increased multiplicatively with salt concentration, reaching a maximum increase of 126.9 nmol/g FW, which was 2.3 times that of the control. This suggests that this endogenous regulation becomes more pronounced under foliar salt stress. MDA accumulation indicates the degree of oxidative damage in plants under stress [38]. Under root salt stress at 0.5 M (approximately 2.9%) [39], the maximum increase in MDA content was only about 4 nmol/g FW. However, under foliar salt stress at 3.0%, the maximum increase in MDA reached 62.31 nmol/g FW. This suggests that when salt ions directly invade leaves, MDA accumulation is highly significant, and the integrity of the membrane system is more severely damaged by peroxidation. Studies have indicated that proline can also act as an effective ROS scavenger, working synergistically with other antioxidant systems to alleviate oxidative stress [36,40]. We also observed a highly significant positive correlation between proline and MDA. Furthermore, proline, together with other carbohydrates, can act as a sugar signal to trigger stress-related responses and serve as a nitrogen and carbon source to aid plant recovery from stress [39,41]. Omics studies have shown that the *P5CS1* and *P5CS2* genes in plants can induce overproduction of proline, thereby enhancing salt tolerance [42].

The stomatal response at the early stage of foliar salt stress is also very rapid. The correlations among Gsw, Pn, growth status, and salt concentration suggest that stomatal inhibition in *V. inermis* reduces its Pn, which limits the supply of energy and materials, leading to inhibition of cell growth and division, impaired leaf and root development, and consequently reduced plant growth. This effect becomes more pronounced with higher salt concentrations, a pattern similar to that observed under root salt stress in wheat [23]. The significant negative correlation between MDA and chlorophyll content suggests that oxidative damage impairs the photosynthetic membrane system and hinders chlorophyll synthesis, thereby inhibiting photosynthesis through non-stomatal factors. Studies have shown that damage to chloroplast structure leads to chlorophyll loss and degradation [43], which explains the sharp decline in chlorophyll content and the progressive yellowing and abscission of older leaves during early stress.

Taken together, these findings suggest that when the external environment changes, plants rapidly sense and respond to stress. However, these rapid responses are not entirely negative. First, because the stress in this experiment was applied directly to leaves as salt spray, the early abscission of older leaves in *V. inermis* helps remove excess salt from the plant. Together with stomatal closure, this minimizes direct salt intrusion and deposition on leaf surfaces. The negative correlation between Gsw and SMC suggests that plants reduce transpiration by decreasing stomatal conductance, thereby limiting water loss and optimizing water use efficiency under salt-induced physiological drought. This response is similar to that under root salt stress [41], although the latter mainly restricts the transport of toxic ions from roots to shoots [44].

### 4.2. Resource Redirection Under Constant Foliar Salt Stress

As foliar salt stress continued and older leaves continued to abscise, the growth status value of *V. inermis* in all stressed groups decreased to a critical threshold around 2.0 between weeks 2 and 4, and fluctuated near this value. From the 5th week of stress onward, plant growth showed a turning point, as evidenced by the successive emergence of new buds on the stems, and the growth status value gradually recovered to the control level. Many short-term root salt stress studies on species including *V. inermis* have not observed this phenomenon [20,23,28,45,46].

Studies on biomass production of *V. inermis* under root salt stress have shown that plants actively reduce their growth rate under salt stress to increase survival [28,37]. This is consistent with the changes in LN and RT observed in our experiment. As stress persisted, Gsw, E, and Pn remained below control levels. The correlations among Gsw, E, Pn, LN, RT, and growth status at the end of the stress period indicate that because photosynthesis is the main source of energy and materials for plant growth and development, the number of new leaves, root development, and the corresponding growth status value in each stressed group remained significantly lower than the control level throughout the experiment. In turn, abscission of older leaves and reduced production of new leaves not only limit salt entry through leaves but also reduce total leaf area and transpirational water loss [47]. Thus, under long-term foliar salt stress, the reduced growth rate of *V. inermis* is also a strategy to reduce metabolic consumption and cope with stress.

At the end of the foliar salt stress period (145 d), chlorophyll content in the 2.0% and 3.0% treatment groups was significantly higher than that in the 1.0% treatment group. This phenomenon has also been observed in short-term root salt stress studies on *V. inermis* [28,37]. This suggests that under moderate to severe salt stress, *V. inermis* may compensate for the decrease in photosynthetic efficiency caused by reduced total leaf area by increasing chlorophyll content, thereby generating energy to mitigate salt damage. However, the positive correlation between MDA accumulation and stress duration indicates that oxidative damage intensifies, especially in the high-salinity groups, which ultimately resulted in chlorophyll content in all stressed plants remaining below the control level.

Notably, although branches above the plastic film in the 2.0% and 3.0% groups were severely desiccated, new buds emerged from the base of the main stem, where they were protected from direct salt spray by the plastic film (Supplementary Figure S4). Leaves that developed under the film were closer to the control group in terms of morphological, physiological, and photosynthetic characteristics than leaves directly exposed to salt spray. This indicates that when part of the plant is stressed, *V. inermis* can redirect growth resources to unstressed tissues. Our field survey on Dongxiang Island also revealed that trees and shrubs planted on windward slopes, which should normally grow upright, instead exhibited withered upper branches under foliar salt stress from sea wind, while branches creeping on the ground continued to sprout and grow. Clearly, this “benefit-seeking and harm-avoiding” strategy, together with reduced growth rate and metabolic consumption, constitutes a resource redirection mechanism that enhances long-term tolerance to foliar salt stress [28].

### 4.3. Trait Plasticity Suggests Adaptation to Foliar Salt Stress

Trait plasticity refers to the ability of an organism to adjust its phenotype in response to environmental conditions without genetic variation [48,49]. Shan’s study [19] reported that under short-term root salt stress, the leaf thickness of *V. inermis* increased approximately 1.5-fold from 0.264 mm to 0.403 mm. In the present experiment, under continuous foliar salt stress, newly developed leaves on the lateral branches of *V. inermis* showed even more pronounced thickening compared with the control, reaching a maximum thickness of 2.011 mm. This trait change persisted throughout the stress period from the emergence of new buds and was significantly positively correlated with salt concentration. We also observed leaf thickening of *V. inermis* in the beach vegetation restoration area on Tangyu Island (Supplementary Figure S1). We found that this thickening was mainly due to elongation of palisade mesophyll cells (Supplementary Figure S5). The thickening of both palisade and spongy tissues in *V. inermis* leaves under salt stress reduces intercellular spaces, thereby limiting CO_2_ conductance through both stomatal and mesophyll pathways and affecting photosynthetic efficiency [50]. This is consistent with the significant negative correlation we observed between photosynthetic indices and LT, suggesting that trait plasticity induced by foliar salt stress also affects photosynthetic efficiency.

Studies have shown that salt damage to protoplasts is closely related to intracellular ion concentration [51]. Some plant cells counteract this by increasing cell volume, thereby maintaining a stable water uptake rate and balancing internal ion levels [40]. In our laboratory experiment, we also found a significant positive correlation between Pro and LT. This suggests that proline helps maintain cell turgor and promotes water influx. Other research has indicated that on the vacuolar membrane, NHXs, which are driven by a proton gradient formed by H^+^-ATPase and H^+^-pyrophosphatase, participate in the transportation of Na^+^, realizing the compartmentation of Na^+^ in the vacuole [52]. These findings also help explain the synergistic increase between LT and LWC in *V. inermis* after two years of planting in Quanzhou Bay. Thus, *V. inermis* enhances its adaptation to long-term salt stress through trait plasticity such as leaf thickening.

After salt stress ceased and irrigation resumed, plant growth, morphology, photosynthesis, and physiological functions gradually recovered to control levels. The photosynthetic efficiency of new leaves was closer to that of the control than that of old leaves, indicating that the trait plasticity of *V. inermis* is highly reversible once stress is removed. This reversibility broadens the plant’s stress tolerance range. Furthermore, previous studies have shown that salt glands and epidermal hairs exist on both leaf surfaces of *V. inermis*, further enhancing its salt tolerance [37].

### 4.4. Application in Vegetation Restoration

Salt tolerance refers to the ability of a plant to maintain normal growth without significant damage when subjected to salt stress [1]. Habitat adaptability refers to the degree of adaptation in growth, development, morphology, physiology, and ecological traits of plants under long-term environmental conditions [53]. After 159 days of foliar salt stress, all *V. inermis* plants maintained a growth status value above 2.0, and there was no significant difference between the 1.0% group and the control group. Based on the integrated changes in growth, morphology, photosynthesis, and physiology, *V. inermis* exhibits strong adaptability to long-term foliar salt stress at concentrations ≤2.0%, and can survive despite severe damage when facing foliar salt stress approaching pure seawater salinity.

In the process of coastal vegetation restoration and coastal shelterbelt construction, the intensity and type of salt stress on plants vary depending on the coastal location and distance from the sea [54]. Particularly on windward slopes at certain elevations that are not submerged by seawater, foliar salt stress from sea wind, salt spray, and wave splash is more intense than root-zone stress. This severely restricts the landscape effect of ecological restoration, yet research attention on this issue remains very limited. The experimental device designed in this study more accurately simulates the foliar salt stress that exists in the field, enables variable control, and effectively prevents root-zone salt stress from affecting the accuracy of the experimental results. This facilitates targeted investigation of salt tolerance in different plant species. The long-term experimental setup allows better observation of plant adaptation and tolerance thresholds under stress. Adding a normal irrigation period after stress cessation enables better evaluation of the plant’s self-recovery capacity when stress is alleviated or removed. This provides guidance for the later management of ecological restoration projects.

Field trials confirmed that at multiple coastal locations in Jinjiang, Quanzhou Bay, most artificially planted *V. inermis* grew well after two years. Their thick stems and well-developed branches enabled them to withstand Typhoon Doksuri in July 2023, demonstrating strong adaptability to various coastal habitats. Notably, *V. inermis* plants at lower elevations and closer to the seashore, lacking a front vegetation barrier, experienced stronger salt stress. In response, these plants developed leaves with higher water content and greater thickness. This is consistent with the indoor experimental results, indicating that trait plasticity is a key adaptive strategy of *V. inermis* to long-term salt stress, and provides useful guidance for screening other salt-tolerant species. These strategies may also be applicable to other woody halophytes. Studies have recommended *V. inermis* as a pioneer species for coastal vegetation restoration in the intertidal zone affected by salt spray and mist. When planted together with *Casuarina equisetifolia*, it can enhance the biodiversity and stability of coastal shelterbelts [55].

## 5. Conclusions

This study employed a self-designed experimental device to accurately simulate the foliar salt stress habitat widely found in coastal areas, while effectively avoiding root-zone interference under controllable conditions. Using a long-term experimental design, we revealed the tolerance and adaptation of *V. inermis* to long-term foliar salt stress. Specifically:

(1) Trait plasticity (e.g., leaf thickening), resource redirection (e.g., reduced growth rate, and new bud emergence in un-stressed parts), and strong recovery capacity together enhance *V. inermis* adaptation to long-term foliar salt stress.
(2) *V. inermis* exhibits strong adaptability to long-term foliar salt stress at concentrations ≤2.0%, and can survive despite severe damage when facing foliar salt stress approaching seawater salinity.
(3) Besides osmotic adjustment, proline accumulation helps alleviate oxidative damage.
(4) Field data demonstrated that leaf thickness and leaf water content were significantly associated with distance from the sea and elevation, thereby validating the salt-adaptation strategies observed under controlled conditions.

Future research should explore the molecular mechanisms underlying trait plasticity and resource redirection, and evaluate the applicability of these adaptive strategies to other coastal woody species for improved restoration outcomes.

## Figures and Tables

**Table 1:** Value of corresponding plant growth status.

Score	Description
5.0	Healthy and vigorous growth with green leaves and profuse branching and foliage
4.5	Minimal yellowing of leaves, slight leaf discoloration
4.0	Slight necrosis at leaf tips, slight desiccation of leaves
3.5	A few yellowing of leaves, pale coloration, drooping leaves
3.0	Less than half necrosis at leaf tips, desiccation, leaf color faded, curled leaves
2.5	More than half yellow leaves, pale coloration, drooping leaves; however, new leaf growth after wilting
2.0	Most necrotic leaf tips, desiccation, drooping leaves, leaf color faded, curled leaves
1.5	Severe yellowing of leaves, pale color, drooping
1.0	Extremely necrosis at leaf tips, desiccation, pale color, curled leaves
0.5	Almost no green leaves
0.0	Dead

**Table 2:** Correlation analysis between indexes of *Volkameria inermis* under foliar salt Stress.

	Salt	Days	Growth	LT	LN	RT	SMC	Pro	MDA	Chl	Pn	E	GSW
**Salt**	1.000	0.000	− 0.782 **	0.887 **	− 0.840 **	− 0.853 **	0.353	0.875 **	0.781 **	− 0.700 **	− 0.798 **	− 0.599 **	− 0.638 **
**Days**		1.000	0.065 *	−0.178	——	——	——	−0.230	0.434 *	−0.004	−0.090	− 0.257 **	− 0.157 *
**Growth**			1.000	− 0.793 **	0.677 **	0.855 **	− 0.565 *	− 0.793 **	− 0.499 *	0.607 **	0.622 **	0.382 **	0.454 **
**LT**				1.000	− 0.797 **	——	——	0.790 **	0.756 **	− 0.588 *	− 0.964 **	− 0.936 **	− 0.936 **
**LN**					1.000	——	——	−0.639 *	−0.614 *	0.767 **	0.796 **	0.764 **	0.764 **
**RT**						1.000	——	——	——	——	——	——	——
**SMC**							1.000	——	——	——	−0.370	−0.204	− 0.227 *
**Pro**								1.000	0.539 **	− 0.498 *	− 0.841 **	− 0.816 **	− 0.846 **
**MDA**									1.000	− 0.448 *	− 0.696 *	− 0.580 *	− 0.580 *
**Chl**										1.000	0.558	0.534	0.496
**Pn**											1.000	0.913 **	0.938 **
**E**												1.000	0.980 **
**GSW**													1.000

Note: ** Significant at the 0.01 level (two-tailed). * Significant at the 0.05 level (two-tailed).

**Table 4:** Correlation analysis between indexes of *Volkameria inermis* in Quanzhou Bay, Jinjiang River.

	Location	Elevation (m)	Plant Height (cm)	LT (mm)	LWC (%)
**Location**	1.000	0.979 **	−0.275	− 0.698 **	− 0.722 **
**Elevation (m)**		1.000	− 0.258	− 0.760 **	− 0.882 **
**Plant Height (cm)**			1.000	0.166	0.523 *
**LT (mm)**				1.000	0.683 **
**LWC (%)**					1.000

Note: ** Significant at the 0.01 level (two-tailed). * Significant at the 0.05 level (two-tailed).

## Data Availability

The original contributions presented in this study are included in the article/Supplementary Materials. Further inquiries can be directed to the corresponding authors.

## Supplementary Materials

The following supporting information can be downloaded at: https://www.mdpi.com/article/10.3390/plants15111756/s1.
