# Supplementary material for: Trait Plasticity, Resource Redirection and Strong Recovery Capacity Enhance Volkameria inermis Tolerance and Adaptation to Long-Term Foliar Salt Stress
Source: Plants (Basel). 2026 Jun 5;15(11):1756. doi: 10.3390/plants15111756 (PMC13259059; doi:10.3390/plants15111756)
Supplement: Supplementary file 1 [file plants-15-01756-s001.zip › plants-4300436-supplementary.pdf]

# Supplementary Materials

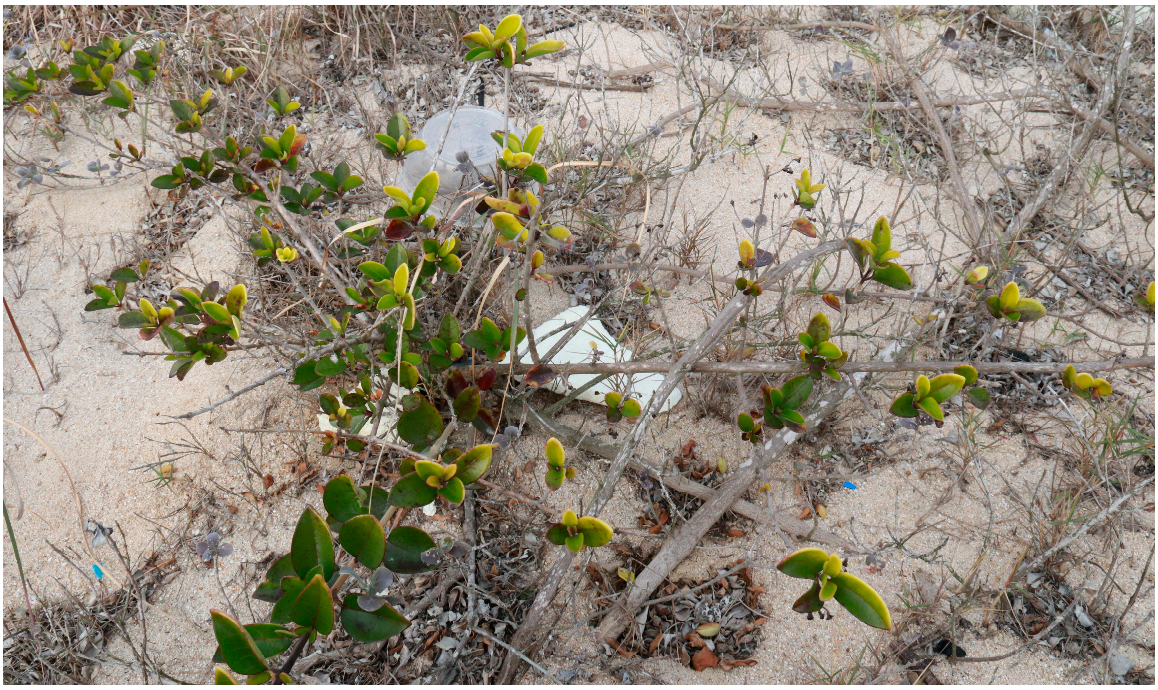

**Figure S1.** The *V. inermis* community on the backshore of Tangyu Island experiences severe salt stress during the winter monsoon due to the lack of tall vegetation on its seaward side. The aboveground parts facing the sea are particularly affected, with leaves exposed to salt mist and wave splash, resulting in significant morphological adaptations such as leaf thickening and succulence.

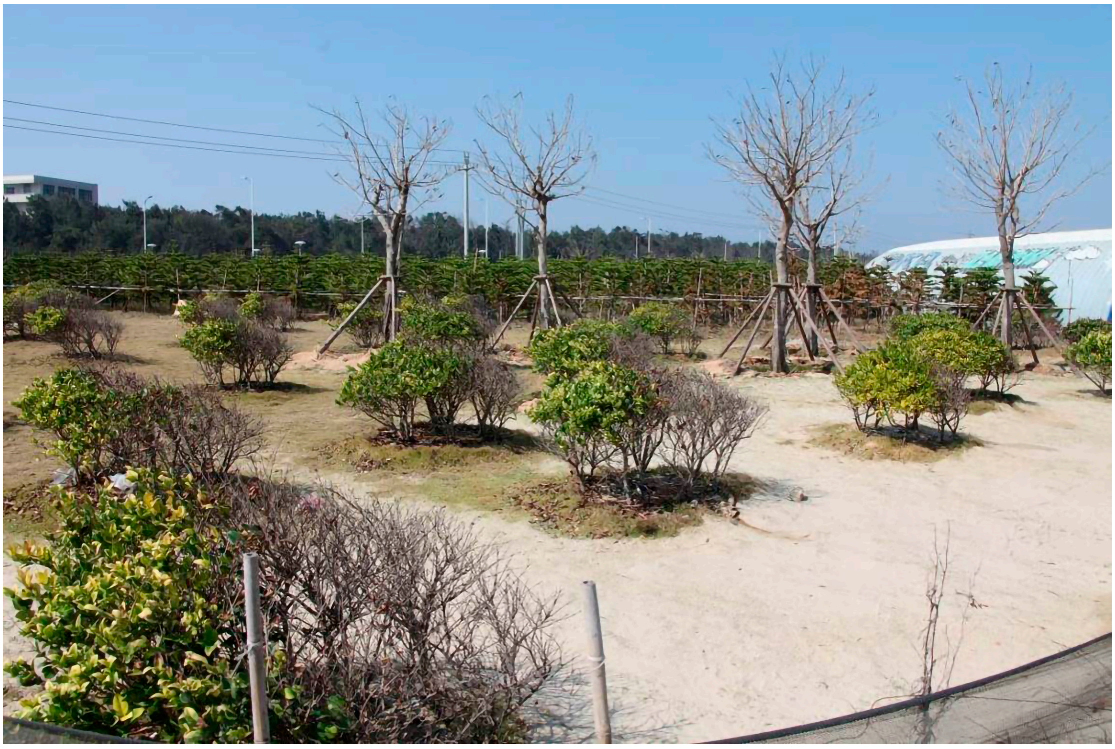

**Figure S2.** In Longfengtou Bay, Pingtan, the seaward branches of planted vegetation withered due to salt spray driven by the winter northeast monsoon and sea waves.

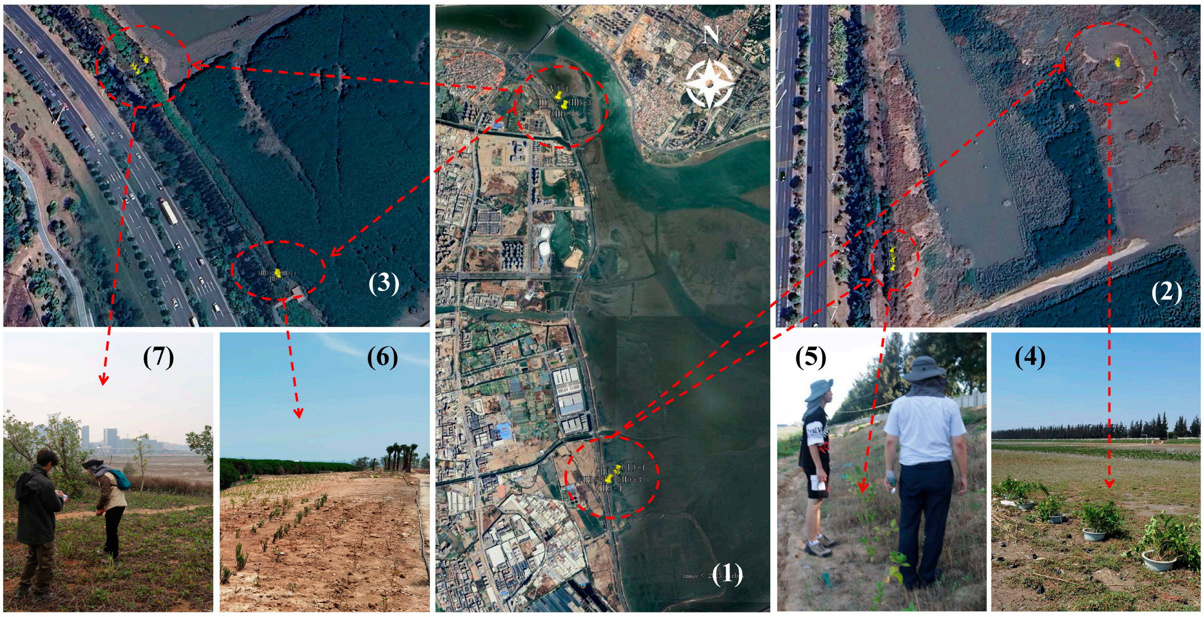


**Figure S3.** Suitability Assessment of *Volkameria inermis* at different locations along the Quanzhou Bay coast, Jinjiang, China. Notes：(1) Satellite map of the *V. inermis* planting area (including two plots, north and south), (2) Satellite image of the southern planting area, (3) Satellite image of the northern planting area, (4) Exposed mudflats in high tide zone mangrove clearings, (5)Toe of the seawall slope affected by spring tides; (6) Seawall slope adjacent to mature mangrove forest margins, (7) seawall slope without mature mangrove forest ahead.

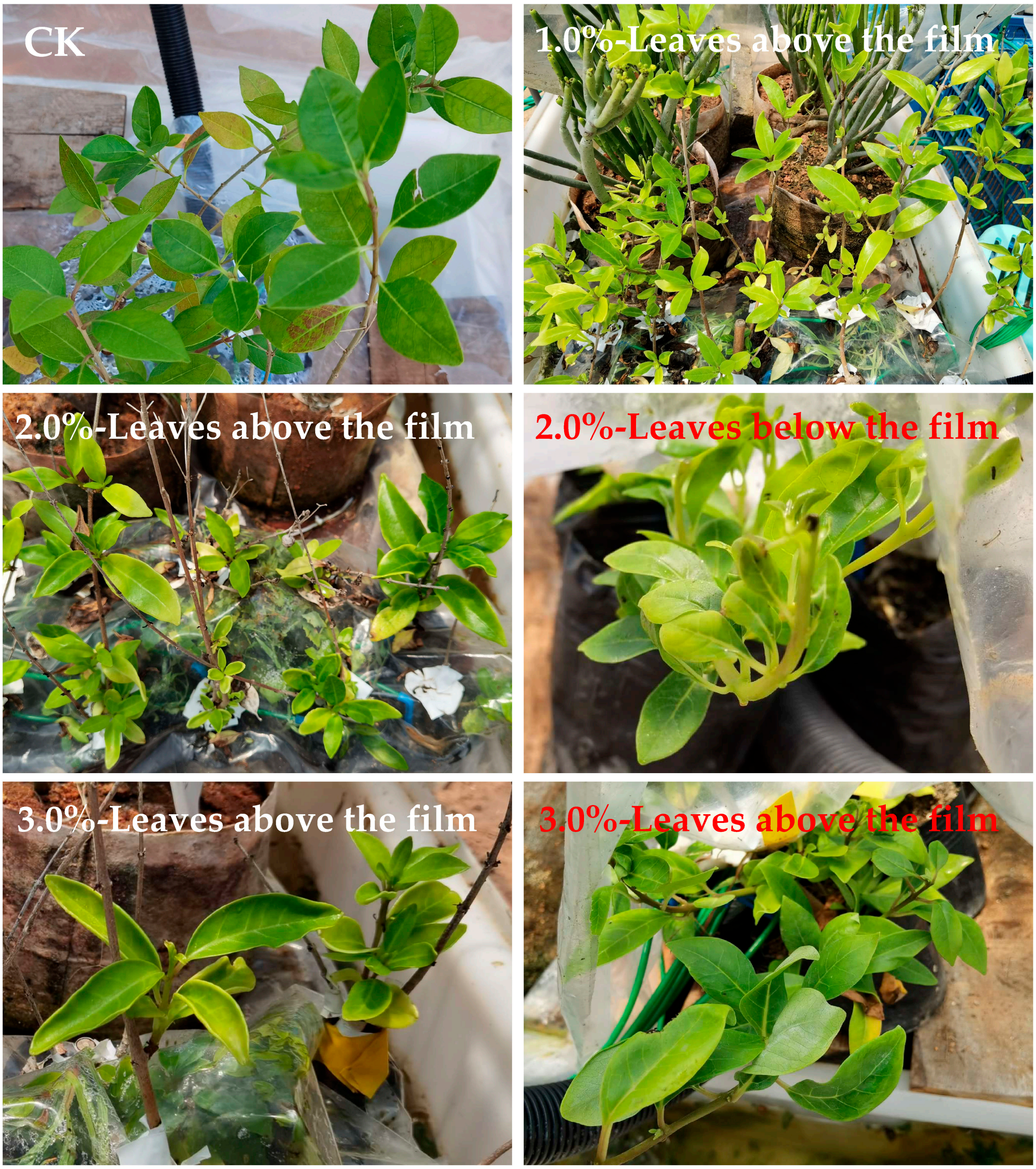


**Figure S4.** Comparison of leaf morphology from different positions of *Volkameria inermis* at day 145 of foliar salt stress.

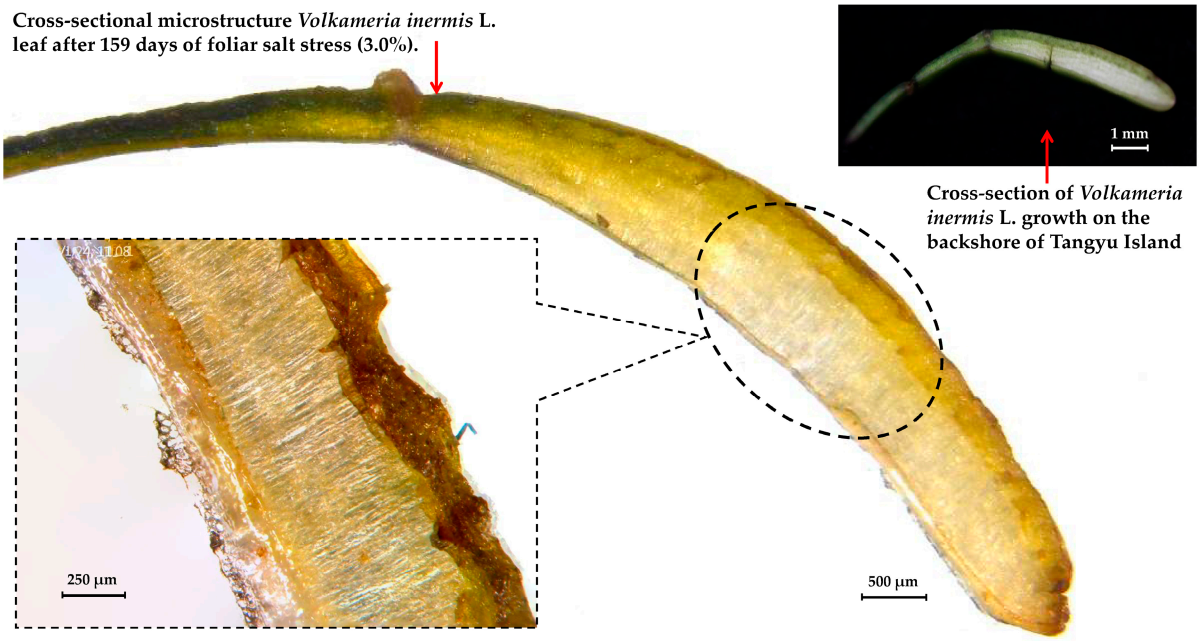


**Figure S5.** Comparison of cross-sectional microstructures perpendicular to the main vein in leaves from stressed *Volkameria inermis* under experimental and field conditions.

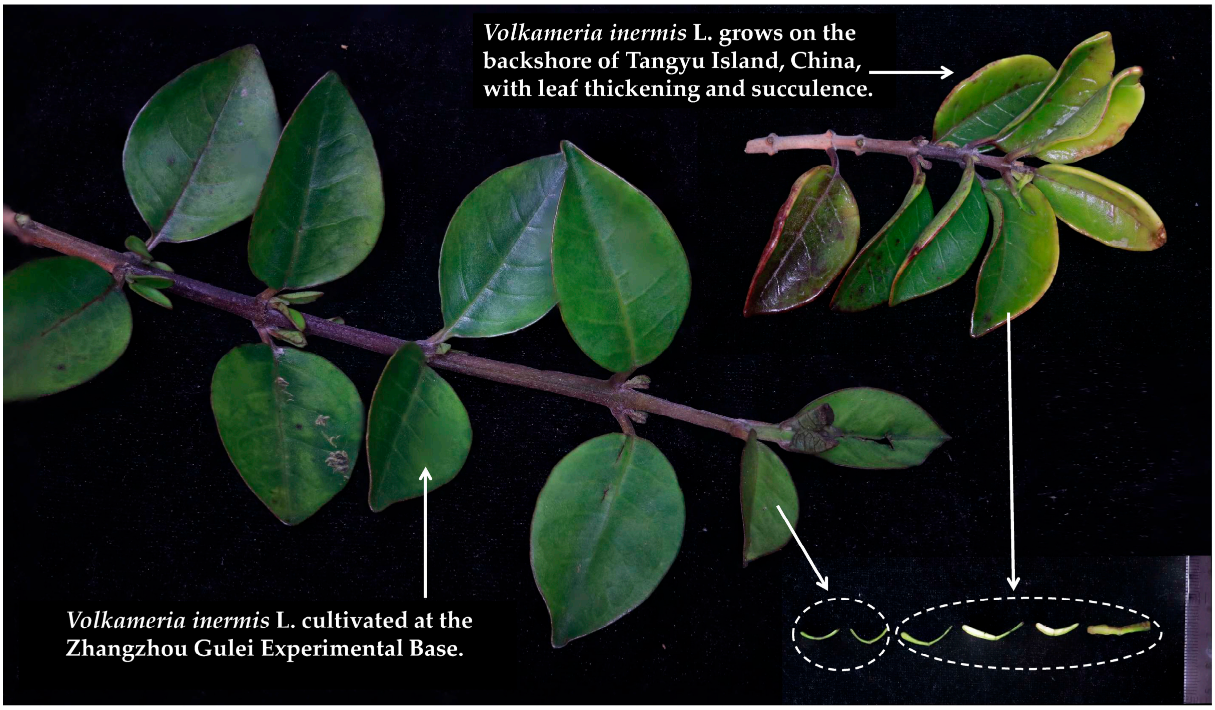


**Figure S6.** Comparison of leaf morphology between stressed *Volkameria inermis* in the field and normally grown plants in the nursery at the Zhangzhou Gulei Experimental Base.
